# Effects of the LPA_1_ Receptor Deficiency and Stress on the Hippocampal LPA Species in Mice

**DOI:** 10.3389/fnmol.2019.00146

**Published:** 2019-06-11

**Authors:** Sara Tabbai, Román Dario Moreno-Fernández, Emma Zambrana-Infantes, Andrea Nieto-Quero, Jerold Chun, Maria García-Fernández, Guillermo Estivill-Torrús, Fernando Rodríguez de Fonseca, Luis Javier Santín, Tiago Gil Oliveira, Margarita Pérez-Martín, Carmen Pedraza

**Affiliations:** ^1^Departamento de Psicobiología y Metodología de las CC, Instituto de Investigación Biomédica de Málaga, Universidad de Málaga, Málaga, Spain; ^2^Sanford Burnham Prebys Medical Discovery Institute, La Jolla, CA, United States; ^3^Departamento de Fisiología y Medicina Deportiva, Instituto de Investigación Biomédica de Málaga, Universidad de Málaga, Málaga, Spain; ^4^Unidad de Gestión Clínica de Neurociencias, Instituto de Investigación Biomédica de Málaga, Hospital Regional Universitario de Málaga, Málaga, Spain; ^5^Unidad de Gestión Clínica de Salud Mental, Instituto de Investigación Biomédica de Málaga, Hospital Regional Universitario de Málaga, Málaga, Spain; ^6^Life and Health Sciences Research Institute (ICVS), School of Medicine, University of Minho, Braga, Portugal; ^7^ICVS/3B’s – PT Government Associate Laboratory, Braga, Portugal; ^8^Departamento de Biología Celular, Genética y Fisiología, Instituto de Investigación Biomédica de Málaga, Universidad de Málaga, Málaga, Spain

**Keywords:** LPA_1_ receptor, LPA species, MALDI-TOFF mass spectrometry, stress, emotions

## Abstract

Lysophosphatidic acid (LPA) is an important bioactive lipid species that functions in intracellular signaling through six characterized G protein-coupled receptors (LPA_1-6_). Among these receptors, LPA_1_ is a strong candidate to mediate the central effects of LPA on emotion and may be involved in promoting normal emotional behaviors. Alterations in this receptor may induce vulnerability to stress and predispose an individual to a psychopathological disease. In fact, mice lacking the LPA_1_ receptor exhibit emotional dysregulation and cognitive alterations in hippocampus-dependent tasks. Moreover, the loss of this receptor results in a phenotype of low resilience with dysfunctional coping in response to stress and induces anxiety and several behavioral and neurobiological changes that are strongly correlated with mood disorders. In fact, our group proposes that maLPA1-null mice represent an animal model of anxious depression. However, despite the key role of the LPA-LPA_1_-pathway in emotion and stress coping behaviors, the available information describing the mechanisms by which the LPA-LPA_1_-pathway regulates emotion is currently insufficient. Because activation of LPA_1_ requires LPA, here, we used a Matrix-Assisted Laser Desorption/ Ionization mass spectrometry-based approach to evaluate the effects of an LPA_1_ receptor deficiency on the hippocampal levels of LPA species. Additionally, the impact of stress on the LPA profile was also examined in both wild-type (WT) and the Malaga variant of LPA1-null mice (maLPA_1_-null mice). Mice lacking LPA_1_ did not exhibit gross perturbations in the hippocampal LPA species, but the LPA profile was modified, showing an altered relative abundance of 18:0 LPA. Regardless of the genotype, restraint stress produced profound changes in all LPA species examined, revealing that hippocampal LPA species are a key target of stress. Finally, the relationship between the hippocampal levels of LPA species and performance in the elevated plus maze was established. To our knowledge, this study is the first to detect, identify and profile LPA species in the hippocampus of both LPA_1_-receptor null mice and WT mice at baseline and after acute stress, as well as to link these LPA species with anxiety-like behaviors. In conclusion, the hippocampal LPA species are a key target of stress and may be involved in psychopathological conditions.

## Introduction

During the past few decades, studies of factors involved in regulating behavior have rarely focused on the role of lipids. However, the study of lipids has received increasing attention, since lipid deregulation is involved in the pathogenesis of nervous system diseases. The lack of studies focusing on lipids is probably due to the scarcity of pertinent tools and the difficulty in analyzing the lipid composition of brain tissue (Bou Khalil et al., 2010). Advances in lipidomics technology have enabled the precise detection, identification and profiling of lipid species in blood and tissues (Rappley et al., 2009) and have allowed researchers to identify how the lipid composition may influence in behavior (reviewed in Miranda et al., 2019), particularly emotional behavior and stress regulation. However, only very few studies have focused on the effects of both acute and chronic stress on the lipid composition or the involvement of lipids in the mechanism regulating mood. According to a systematic study, the metabolism of sphingolipids and phospholipids is significantly altered by stress or exogenous corticosterone administration in several key regions controlling emotion, such as the prefrontal cortex and hippocampus (Oliveira et al., 2016). Alternatively, chronic unpredictable stress exposure induces changes in relative cellular levels of both signaling and structural lipids in the brain, including increased phosphatidylcholine (PC) and phosphatidylethanolamine (PE) contents and decreased phosphatidylinositol (PI), phosphatidic acid (PA) and cardiolipin (CL) contents (Faria et al., 2015). Several lines of evidence implicate changes in lipid metabolism in depression (DeMar et al., 2006; Müller et al., 2015; Ong et al., 2016). Additionally, changes in lipid levels have been identified in patients with major depressive disorder (Lin et al., 2010). In fact, a phospholipid hypothesis of depression has been proposed (Horrobin and Bennett, 1999), and lipids are one target of antidepressant treatments (Lee et al., 2009).

Among lipids, recent data reveal lysophophatidic acid (LPA) as an important lipid signaling molecule that controls behavior (Santin et al., 2009; Castilla-Ortega et al., 2010, 2014, 2016; Orio et al., 2013; Pedraza et al., 2014; Mirendil et al., 2015; Yamada et al., 2015; Moreno-Fernández et al., 2018a; Ladrón de Guevara-Miranda et al., 2019; Sánchez-Marín et al., 2018). LPA is an endogenous, simple, bioactive phospholipid with several biological functions (Yung et al., 2014). LPA species include both saturated fatty acids [palmitic acid (16:0) and stearic acid (18:0)] and unsaturated fatty acids [oleic (18:1), linoleic (18:2) and arachidonic acids (20:4)] (Sano et al., 2002; Sugiura et al., 2002) with different biological activities (Hayashi et al., 2001; Yoshida et al., 2003; Aikawa et al., 2015), suggesting that multiple synthetic pathways participate in LPA production. Thus, because saturated and unsaturated fatty acids predominantly bind to the sn-1 and sn-2 positions, respectively, phospholipase A_1_ (PLA_1_) and phospholipase A_2_ (PLA_2_) are involved in the production of different LPA species. Moreover, the levels of autotaxin (ATX), an enzyme with lysophospholipase D (LysoPLD) activity, strongly correlate with LPA concentrations (Watanabe et al., 2007; Nakamura et al., 2008), and its depletion in serum and plasma abolishes the LPA production. Thus, a reasonable hypothesis is that this enzyme is responsible for producing LPA (Tabuchi, 2015). LPA species exhibit different biological activities by signaling through different LPA receptors. Six G protein-coupled receptors, LPA_1-6_, have been identified as mediating LPA signaling (Choi et al., 2010; Kihara et al., 2015), although two additional receptors (GPR87 and P2Y10) have recently been identified (Tabata et al., 2007; Oka et al., 2010).

Although the participation of the LPA receptors, particularly the LPA_2_ (Trimbuch et al., 2009; Schneider et al., 2018), LPA_3_ (Uchida et al., 2014; Ueda et al., 2018) and LPA_5_ receptors (Callaerts-Vegh et al., 2012; Tsukahara et al., 2018) on behavior regulation may not be ruled out, in the brain, LPA appears to function as a regulatory molecule that mainly signals through the LPA_1_ receptor (Choi et al., 2010; Chun et al., 2013). The brain distribution of the LPA_1_ receptor in emotion-processing regions and the use of LPA_1_ receptor null mice, which provide essential information on the putative function of the LPA in the brain, have revealed a role for the LPA-LPA_1_-receptor pathway in regulating the neurobiological variables involved in affective states (Santin et al., 2009; Castilla-Ortega et al., 2011; Pedraza et al., 2014; Moreno-Fernández et al., 2017, 2018a, b). In fact, animals lacking the LPA_1_ receptor exhibit emotional dysregulation, impaired extinction of aversive memories (Pedraza et al., 2014), an anxious phenotype (Santin et al., 2009; Castilla-Ortega et al., 2010; Moreno-Fernández et al., 2017, 2018a), cognitive alterations in hippocampus-dependent tasks (Santin et al., 2009; Castilla-Ortega et al., 2010, 2011) and dysfunctional coping in response to chronic stress (Castilla-Ortega et al., 2011). Thus, recent evidence suggests a role for the LPA_1_ receptor in regulating the effects of stress on the hippocampus, and the lack of LPA_1_ signaling confers vulnerability to chronic stress, which precipitates hippocampal pathology (Castilla-Ortega et al., 2011; García-Fernández et al., 2012) and exacerbates the stress-induced neuroplastic changes, such as reduced adult neurogenesis, increased apoptosis (Castilla-Ortega et al., 2011) and enhanced oxidative stress (García-Fernández et al., 2012). Data from both genetic and pharmacological approaches are convergent and provide evidence supporting the hypothesis that the LPA_1_ receptor plays an essential role in regulating emotions and mood. The inactivation of this receptor induces depression-like behaviors with an agitation component and is linked to functional changes in key brain regions involved in the stress response and emotional regulation (Pedraza et al., 2014; Moreno-Fernández et al., 2017, 2018a,b).

Ongoing studies are beginning to reveal mechanisms by which the LPA_1_ receptor regulates emotion and the stress response. However, much less is known about the changes in LPA species and their involvement in regulating emotion. Here, we used a MALDI-TOF mass spectrometry-based approach to evaluate the impact of an LPA_1_ receptor deficiency on the hippocampal levels of specific LPA species (LPA 16:0, LPA 18:0, LPA 18:1, and LPA 18:2). Additionally, the effect of stress (induced by restraint or the exposition to a moderate acute stress model, the elevated plus maze (EPM)), on the LPA profiles of both wild-type and maLPA_1_-null mice and their relationship with behavioral performance were examined. Finally, because corticosterone (CORT) is one of the principal mediators of the impact of stress on the brain and behavior, the blood levels of this hormone were measured.

## Materials and Methods

### Animals

**FIGURE 1:**
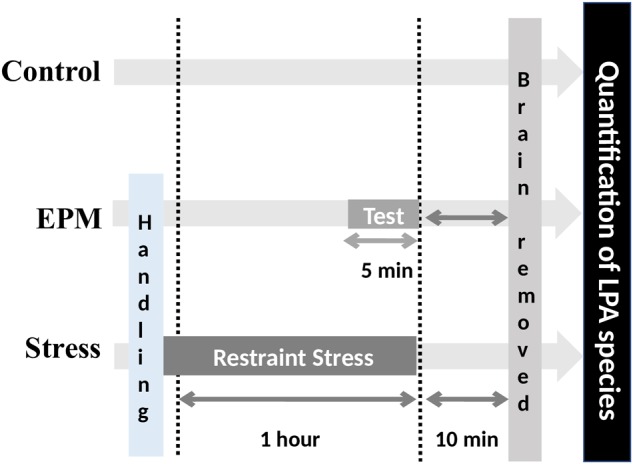
Experimental procedure. Effects of the three environmental conditions (control, stress induced by EPM or restraint stress) on hippocampal LPA levels in both WT and maLPA1-null mice.

In this study, 36 male mice aged 3 months (15 wild-type mice and 21 mice lacking the LPA_1_ receptor (maLPA_1_-null mice; the Malaga variant of LPA1-null mice) were used. The litter number was 14 and the average litter size was 7 (ranged between 4 and 9). All experiments were performed using mice with a mixed C57BL/6 × 129X1/SvJ background. Trials were conducted on age-matched male wild-type and maLPA_1_-null homozygous littermates that were approximately 3 months old. The maLPA1-null mouse (termed maLPA1-*Málaga* variant of the LPA1 null-mouse, was spontaneously derived during the original colony (Contos et al., 2000) expansion by crossing heterozygous foundational parents (maintained in the original hybrid C57BL/6J6 × 1291/SvJ background). MaLPA_1_-null mice arising from the colony Malaga variant of LPA1-null mice were developed in our laboratory and have been described in previous studies (Matas-Rico et al., 2008; Estivill-Torrus et al., 2008). Animals were collectively housed in same-genotype groups and same-experimental groups (Animal Resource Center at the University of Malaga) in a room with a temperature of 21 ± 2°C and 55 ± 5% relative humidity with a 12 h light/dark cycle and had free access to a standard laboratory diet and tap water. The animals were distributed randomly into a control group (*N* = 5 wild-type; *N* = 5 maLPA_1_-null mice); animals that performed the elevated plus maze (EPM) (*N* = 5 wild-type; *N* = 8 maLPA_1_-null mice) and a stress group subjected to restraint stress for 1 h (*N* = 5 wild-type; *N* = 8 maLPA_1_-null mice) (Figure 1: Experimental procedure).

Procedures were approved by the Ethics Committee of Malaga University (CEUMA 2013-0008-A/CEUMA: 1-2015- A, 08-7- 15-273) and Junta of Andalucia (08-7-15-273) and were performed in compliance with European animal research laws [European Communities Council Directives 2010/63/UE, 90/219/CEE, Regulation (EC) No. 1946/2003] and Spanish National and Regional Guidelines for Animal Experimentation and Use of Genetically Modified Organisms (Real Decreto 53/2013, Ley 32/2007, and Ley 9/2003, Real Decreto 178/2004, and Decreto 320/2010).

### Stress Procedure

#### Behavioral Test

The elevated plus maze (EPM), a moderate acute stress model and widely used behavioral test for rodents, was employed (Carobrez and Bertoglio, 2005). The EPM is a simple method for assessing the anxiety responses of rodents (Pellow et al., 1985; Walf and Frye, 2007). However, it may be considered an aversive test that induces moderate increases in stress hormone levels (Mendes-Gomes et al., 2011; Fodor et al., 2016). Based on these findings and taking into account that the luminosity conditions used in our study (90–100 lux) may have anxiogenic properties (reviewed in Violle et al., 2009), the test was used as a mild source of acute stress (Filgueiras et al., 2014) and to assess the possible relationship between the levels of various LPA species and behavioral performance. The EPM consists of two open arms (30 cm long × 5 cm wide; 90–100 lux) and two enclosed arms (30 cm long × 5 cm wide × 15 cm high walls; 25–30 lux) connected to a common central platform (5 cm × 5 cm) and is elevated 38.5 cm above the floor. For the test, animals were removed from the cage and placed at the junction of the open and closed arms, facing the open arm. At the end of the 5-min test, animals were removed from the EPM and placed in the home cage. Using a video tracking system (Ethovision XT, Noldus, Wageningen, Netherlands), the total time spent in each arm and the number of total arm entries were assessed. Additionally, an anxiety index was calculated using the following equation (Cohen et al., 2004; Contreras et al., 2014; da Costa Estrela et al., 2015) (1):

(1) $$\begin{array}{c} Axiety\;index\;=1-\;\left[\frac{(\frac{open\;arms\;time\;(in\;seconds)}{test\;duration\;\left(in\;seconds\right)})\;+(\frac{number\;of\;open\;arms\;entries}{total\;numbers\;of\;entries\;})}{2}\right] \end{array}$$

This index, for which the values range from 0 to 1, integrates several variables evaluated in the EPM (Contreras et al., 2014). A score closer to 1 indicates greater anxiety-like behaviors.

Because the extremes of the open arms (distal ends) are the most anxiogenic region of the test, the time in seconds, the number of entries, the latency to the first distal end entry and the percentage of time that animals remain in the distal ends relative to the total time spent in the open arms were evaluated. Locomotion [distance traveled (cm)] and velocity (cm s - 1) were also recorded. Moreover, ethological patterns exhibited by the animals (freezing behavior, rearing, grooming, risk assessment, and head dipping) were scored by two researchers blind to animal identity, with an inter-rater reliability measures over 80%. *Freezing* was measured as time spent by the animal completely immobile during the EPM test. *Grooming* is one of the most frequently observed behaviors in awake rodents and it was assessed as repetitive, self-directed and sequentially patterned behaviors of hygiene and self-care. *Risk assessment* was defined as stretch-attend postures toward the open arms of the EPM. *Head dipping* time was measured as the seconds the mice spent protruding the head over the edge of the open arms (Nunes-de-Souza et al., 2002; Bibancos et al., 2007; Schneider et al., 2011, Sorregotti et al., 2013).

#### Restraint Stress

Animals were submitted to an acute stress procedure by restraint. Acute restraint is an uncontrollable stress condition that induces several emotional and autonomic responses (Reis et al., 2011). For this purpose, animals were placed in a 50 ml clear polystyrene conical centrifuge tube modified with air holes for ventilation (Castilla-Ortega et al., 2011; García-Fernández et al., 2012; Zimprich et al., 2014) for 1 h. After restraint procedure mice were placed back into their transport cages. Control mice remained undisturbed in their home cages.

### LPA Extraction From Hippocampal Tissues

Ten minutes after completing the stress procedure or the behavioral test, all animals were decapitated. Brains were removed and the hippocampus was macrodissected, collected in a tube, drop-frozen in liquid nitrogen and subsequently stored at -80°C until further analyses of LPA species. The average wet weight of the isolated unilateral hippocampus from each mouse was approximately 10 mg of tissue weight.

Lysophosphatidic acid was extracted from the hippocampal tissues using a modified version of previously described methods (Tanaka et al., 2004; Morishige et al., 2010; Ma et al., 2013). First, the isolated hippocampus was resuspended in 200 μl of O-vanadate [100 mM] and EDTA [1 mM] and homogenized. Subsequently, 1 ml of acetone with 0.5 nmol of internal standard (17:0 LPA) was added. The resulting pellet was washed twice with 0.5 ml of acetone and dried under a nitrogen stream. Then, 380 μl of CHCl_3_, MeOH, and H_2_O (1:2:0.8) were added and centrifuged for 5 min for two repetitions. A solution of 0.4 ml of CHCl_3_, 0.4 ml of KCl, and 2 μl of ammonia (28%) was added to the supernatant. The mixture was centrifuged at 1300 × *g* for 5 min and the CHCl_3_ phase was removed. The aqueous phase was washed 4 times with a solution composed of 0.8 ml of CHCl_3_ and MeOH (17:3). Afterward, 50 μl of Phos-tag [1 mM] (Monoisotopic68Zn^2+^-Phos-tag (Phos-tag^®^) obtained from the NARD Institute Ltd. (Hyogo, Japan) and 0.8 ml of a CHCl_3_ and MeOH solution (17:3) were added. The mixture was centrifuged at 1300 × *g* for 5 min and the CHCl_3_ phase was collected. The aqueous phase was washed twice with 0.8 ml of the CHCl_3_ and MeOH solution (17:3). The CHCl_3_ phase was collected and dried under a nitrogen stream. Finally, the LPA product was dissolved in 100 μl of MeOH/NH_4_OH (0.1%).

### LPA Analysis Using MALDI TOFMS

Tissue phospholipids were analyzed using an μtraflex MALDI-TOF mass spectrometer (MALDI-TOF MS) (Bruker Daltonics, Bremen, Germany). To this end, 1 μl was removed from 100 μl of LPA in a MeOH/NH_4_OH (0.1%) solution and mixed with 1 μl of a THAP solution (10 mg/ml) in acetonitrile. The mixture was spotted on an MALDI plate (Bruker Daltonics, Bremen, Germany). After drying, the sample was washed with water and dried again. Mass spectrometry was performed in the positive mode using an acceleration voltage of 25 kV. The laser energy was 30 –70% (3.0 – 7.0 μJ) with a repetition rate of 10 Hz. The mass spectra were calibrated externally using a peptide calibration standard (Bruker Daltonics, Bremen, Germany). Each spectrum was produced by accumulating data from 3000 or 6000 consecutive laser shots.

For calibration curves, LPA standards [16:0, 18:0, 18:1, 18:2, and 20:4, which were purchased from Avanti Polar Lipids, Inc. (Alabaster, AL)] and an internal standard (17:0 LPA, purchased from Avanti Polar Lipids) were mixed at different molar ratios and dissolved MeOH/NH_4_OH (0.1%). Then, 50 μL of Phos-tag [1 mM] were added. The resulting mixture was analyzed using the aforementioned procedure for determining tissue phospholipid levels. The concentrations in hippocampal tissues were determined based on the ratio of the intensity of Phos-tag-linked LPA to the internal standard 17:0 LPA linked to Phos-tag using the equations for calibrations curves for all LPA standards. Since the level of 20:4 LPA in the hippocampus was below the detection limit, this species was not subjected to further analysis. Total LPA concentrations, which represents the sum of all LPA species determined, except for 20:4 LPA, were also calculated. Concentrations of LPA and LPA species are reported as pmol/mg of hippocampal tissue.

Additionally, lipid concentrations of each species were normalized to the concentration of all LPA species detected in the hippocampus (Oliveira et al., 2016) and the data are presented as the mean percentages (see equation 2). These values may be interpreted as reflecting the relative abundances of LPA species analyzed in the hippocampus to total LPA species.

(2) $$\begin{array}{c} \%\;LPA\;species=100^{*}\;\frac{\left[LPA\;species\right]}{\left(\left[LPA\;16:0]+[LPA\;18:0]+[LPA\;18:1]+[LPA\;18:2\right]\right)} \end{array}$$

### Corticosterone Experiment

Mice were decapitated 10 min after completing the behavioral test or the 1 h restraint procedure, and trunk blood was collected and allowed to clot to obtain serum samples. For the measurement of corticosterone levels, blood samples were centrifuged and the supernatant stored at -80°C. Serum corticosterone levels were measured using a commercially available enzyme immunoassay kit, with a sensitivity of ca. 27.0 pg/ml, according to the manufacturer’s instructions (Assay Designs/Stressgen, Ann Arbor, MI, United States).

### Statistical Analysis

All results are presented as means ± SEM; *p* < 0.05 was considered statistically significant.

#### Behavioral Tests

The duration of behaviors exhibited by animals of both genotypes during the EPM were analyzed using Student’s *t*-test (*t*). Levene’s test was used to test the assumption of homogeneity of variance. Welch’s *t*-test (t_w_) was performed when two samples had unequal variances.

#### LPA Species Quantification

First, the impact of the LPA_1_ receptor deficiency on the LPA profile was assessed. Because the equality of variance was satisfied, Student’s *t*-test was used to examine differences for total LPA concentrations and the concentrations of every LPA species between WT and maLPA_1_-null mice.

One-way ANOVA followed by the *post hoc* Fisher’s least significant difference test (LSD) were used to assess the effects of stress on LPA species. Then, the effects of interaction between the LPA_1_ receptor deficiency and stress were examined using a factorial ANOVA with two factors: genotype (wt or maLPA_1_-null mice) and environmental treatment (control, EPM or restraint stress) followed by *post hoc* LSD analyses when required.

These analyses were performed for both the concentration and percentage of relative abundance.

#### Corticosterone Analysis

Differences in CORT levels between genotypes and environmental treatments (control, EPM and restraint stress) were analyzed using a factorial ANOVA and *post hoc* Fisher’s least significant difference (LSD) analysis.

#### Principal Component Analysis

A principal component analysis (PCA) was subsequently performed on the hippocampal LPA species concentrations and behaviors exhibited by animals in the EPM to obtain insights into the LPA species that were associated with behavioral performance. This analysis allowed us to characterize the lipid profile associated with behaviors by reducing the full, multidimensional set of LPA species data to a smaller set of dimensions underlying behavior.

We used the Kaiser-Meyer-Olkin (KMO) test to test whether the PCA met the statistical adequacy criteria (Balaban et al., 2014). Then, because the component or factor scores represent the relative contribution or weight of each loading pattern for each case, Student’s *t*-test was used to determine differences in each loading pattern between WT and maLPA1-null mice.

Only probabilities of *p* ≤ 0.05 were considered significant. For clarity and brevity, only relevant results from these statistical analyses are reported.

## Results

### Behavioral Test

**FIGURE 2:**
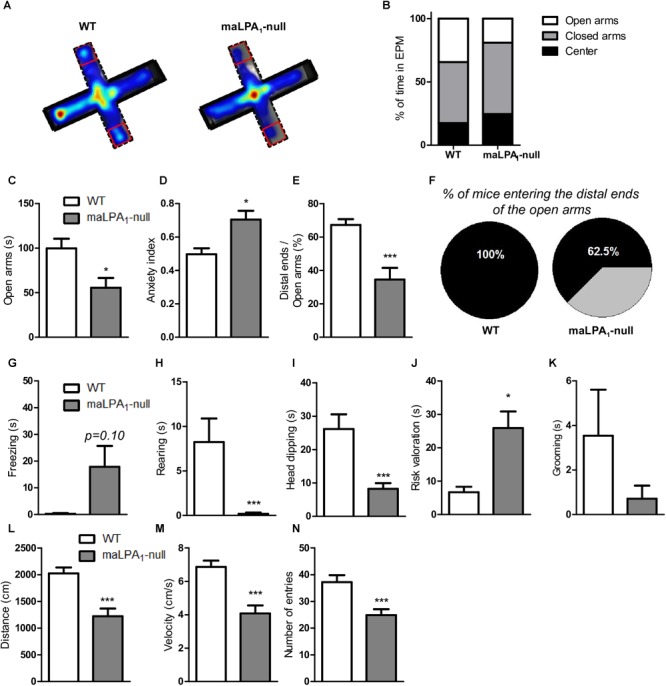
Behavioral test using the EPM. **(A)** Composite map of all paths traveled during the EPM test by each genotype. The maps are presented as “heat maps” indicating mean dwell times according to a color code. Warmer colors indicate high dwell times in the different compartments of the EPM and cooler colors indicate low dwell times. **(B)** Percentage of time spent in the EPM divided by percentage of time spent in the open arms, closed arms and center. **(C)** Time spent in the open arm. **(D)** Anxiety index. **(E)** Number of entries in the distal ends relative to the total number of entries in the open arms. **(F)** Percentage of time animals remained in the distal ends relative to the total time spent in the open arms. Ethological parameters: **(G)** freezing, **(H)** rearing, **(I)** head dipping, **(J)** risk assessment, **(K)** grooming, **(L)** distance traveled in the maze, **(M)** velocity, and **(N)** number of entries into each arm. ^∗^*p* < 0.05, ^∗∗∗^*p* < 0.0005 maLPA1-null mice with respect to WT animals.

In the EPM, maLPA_1_-null mice showed an anxiogenic phenotype, consistent with previous reports (Figure 2). The maLPA_1_-null mice spent significantly less time in the open arms [t(11) = 2,68; *p* ≤ 0.05] (Figure 2B,C) and showed a reduced number of open arm entries [t(11) = 4,74; *p* ≤ 0.0001]. In fact, a significantly higher anxiety index was observed in mice lacking the LPA_1_ receptor [t(11) = -2,82; *p* ≤ 0.01] (Figure 2D). Moreover, maLPA_1_-null mice exhibited fewer entries in the distal end [t(11) = 3.53; *p* < 0.005], a longer latency to the first distal end entry [t(11) = -2.54; *p* < 0.05] and spent less time in the distal ends relative to the total time spent in the open arms [t_w_(11) = 4.18; *p* < 0.005]. Distance traveled [t(11) = -3.95; *p* ≤ 0.005], average velocity [t(11) = 4.10; *p* ≤ 0.001] and total number of entries [t(11) = 3.53; *p* < 0.005] in the EPM were also reduced in maLPA_1_-null mice, indicating, as previously observed (Santin et al., 2009), less exploratory activity than WT animals. However, the time spent in the open and closed arms did not correlate with the distance traveled by any genotype (*r* = -0.55; *p* > 0.5 and *r* = 0.41; *p* < 0.05 for open and closed arms, respectively, in WT mice and *r* = -0.15; *p* > 0.05 and *r* = 0.21; *p* > 0.5 for open and closed arms, respectively, in maLPA1-null mice). Regarding ethological parameters, the lack of the LPA_1_ receptor induced a significant increase in the risk assessment behavior [t_w_(11) = -3.67; *p* ≤ 0.01] and decreased head dipping [t_w_(11) = 3.80; *p* ≤ 0.001] and rearing behaviors [t_w_(11) = 3.04; *p* ≤ 0.005]. The maLPA_1_-null mice also spent more time freezing and less time grooming, but the differences were not statistically significant [t(11) = -1.74; *p* = 0.10 and t(11) = 0.70; *p* = 0.13] for freezing and grooming, respectively (Figure 2).

### LPA Species Quantification

**FIGURE 3:**
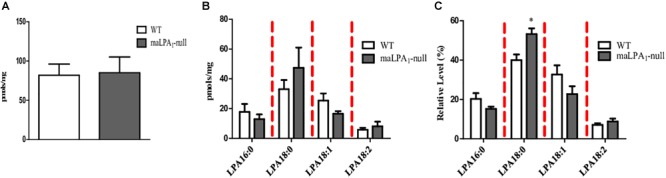
Hippocampal LPA concentrations in both WT and maLPA1-null mice. No differences in the hippocampal concentrations of **(A)** total LPA and **(B)** LPA species were observed between genotypes. **(C)** The LPA receptor deficiency slightly modified the LPA profile by altering the percentage of relative abundance of 18:0 LPA (^∗^*p* ≤ 0.05 maLPA1 compared with WT mice). LPA 16:0; 18:0; 18:1, and 20:4 species [palmitic acid (16:0) and stearic acid (18:0)] [oleic (18:1), linoleic (18:2), and arachidonic acids (20:4)].

The LPA_1_ receptor deficiency did not induce changes in the total LPA concentrations [t(8) = -0.12, *p* > 0.05] (Figure 3A) or the concentrations of any LPA species examined in the hippocampus [t(8) = 0.76, *p* > 0.05; t(8) = -0.7, *p* > 0.05; t(8) = 1.7, *p* > 0.05; t(8) = -0.96, *p* > 0.05 for 16:0; 18:0; 18:1, and 18:2 LPA, respectively] (Figure 3B). Regarding the percentage of relative abundance, the hippocampus of maLPA_1_-null mice presented only minor alterations, with increased levels of 18:0 LPA [t(8) = -3.51, *p* = 0.007], but not inducing changes in the percentage of relative abundance of any of the other species assessed [t(8) = 0.20, *p* > 0.05; t(8) = 0.5, *p* > 0.05; t(8) = 0.12, *p* > 0.05; t(8) = 0.36, *p* > 0.05 for 16:0; 18:0; 18:1, and 18:2 LPA, respectively] (Figure 3C). In both WT and maLPA_1_-null mice, 18:0 LPA was the most abundant LPA species in hippocampus. The second most abundant LPA species was 18:1 LPA. Notably, 20:4 LPA was the least abundant hippocampal species in both genotypes, causing a detection error, and thus it was not subjected to any further analyses (Figure 3B). Based on these data, the LPA_1_ receptor deficiency is not associated with major changes in the lysophopholipid profile in the hippocampus.

**FIGURE 4:**
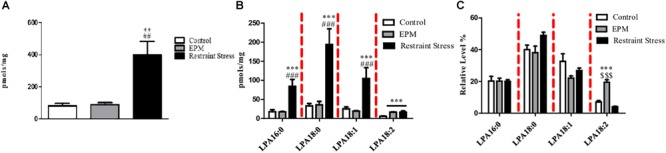
Effects of stress on hippocampal LPA concentrations. Restraint stress produced profound changes in **(A)** the hippocampal concentrations of total LPA and **(B)** all LPA species examined. **(C)** Restraint stress did not affect the percentage of relative abundance, but the EPM modified the profile of LPA species analyzed in the hippocampus. ^∗∗^*p* ≤ 0.001 and ^∗∗∗^*p* ≤ 0.0005 compared with the control group; ^##^*p* ≤ 0.001 and ^###^*p* ≤ 0.0005 compared with the EPM; ^$$$^*p* ≤ 0.0005 compared with restraint stress. LPA 16:0; 18:0; 18:1, and 20:4 species [palmitic acid (16:0) and stearic acid (18:0)] [oleic (18:1), linoleic (18:2) and arachidonic acids (20:4)].

Nevertheless, upon exposure to restraint stress, we observed a gross perturbation in the concentrations of both total LPA and LPA species in the hippocampus (Figure 4A,B). One-way ANOVA revealed effects of stress on the total LPA concentration [*F*(2,12) = 11.66, *p* ≤ 0.01] and the concentrations of all LPA species examined [*F*(2,12) = 12.09; *p* ≤ 0.001 for 16:0 LPA; *F*(2,12) = 14.03; *p* ≤ 0.001 for 18:0 LPA; *F*(2,12) = 13.16; *p* ≤ 0.001 for 18:1 LPA *F*(2,12) = 9.83; *p* ≤ 0.005 for 18:2 LPA]. The *post hoc* analysis showed an increase in the total LPA and LPA species concentrations following restraint stress (LSD; *p* ≤ 0.05). However, behavioral stress (EPM) only affected the concentration of the 18:2 LPA species (LSD: *p* = 0.02). Regarding the effects of stress on the percentage of relative abundance, stress altered the hippocampal LPA profile by significantly increasing the relative abundance of 18:2 LPA [*F*(2, 11) = 52.90; *p* ≤ 0.0001; LSD *p* ≤ 0.005]. Nevertheless, restraint stress did not alter the hippocampal LPA profile (LSD; *p* > 0.05) (Figure 4C). Taking into account all data, restraint stress induced important changes in the hippocampal LPA concentrations (Figure 4A,B). However, EPM modified the relative LPA species abundance (Figure 4C).

**FIGURE 5:**
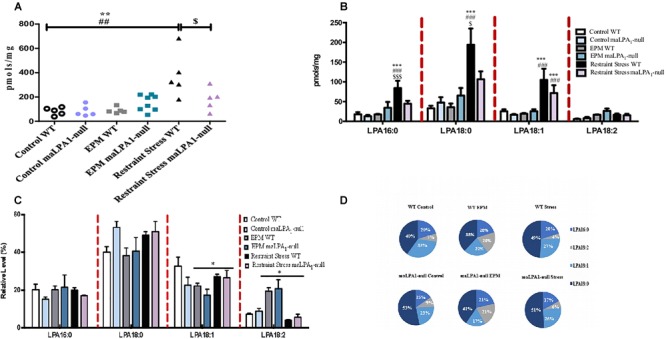
Effects of stress and the LPA_1_ receptor deficiency on hippocampal LPA concentrations. Stress exerted the greatest effects on **(A)** the hippocampal concentrations of total LPA and **(B)** LPA species in WT mice than in maLPA_1_-null mice. **(C)** Regarding the percentage of relative abundance, the effects of stress were only observed on LPA 18:1 and LPA 18:2. **(D)** Graphic representation of the percentage of relative abundance. ^∗∗∗^*p* ≤ 0.0005 compared with the control; ^###^*p* ≤ 0.0005 compared with the EPM; ^$^*p* ≤ 0.05 and ^$$$^*p* ≤ 0.0001 compared with maLPA1-null mice. ^∗^*p* ≤ 0.05 compared with control groups. LPA 16:0; 18:0; 18:1, and 20:4 species [palmitic acid (16:0) and stearic acid (18:0)] [oleic (18:1), linoleic (18:2), and arachidonic acids (20:4)].

Because maLPA_1_-null exhibit an increased vulnerability to stress (Castilla-Ortega et al., 2011), the effects of both stress and the LPA_1_ receptor deficiency on the hippocampal LPA profile were examined (Figure 5). A genotype x stress effect was observed on the total LPA concentration (F (2,27) = 3,77, *p* ≤ 0.05) and in the concentrations of the 16:0 [*F*(2, 27) = 3.87; *p* ≤ 0.05] and 18:0 LPA species [*F*(2, 27) = 4.47; *p* ≤ 0.05]. The *post hoc* analysis revealed significant increases in the total LPA concentration in both genotypes following restraint stress (*p* ≤ 0.00001 WT mice with restraint stress versus WT mice under control conditions and WT mice subjected to the EPM; *p* ≤ 0.05 maLPA_1_-null mice versus maLPA_1_ mice under control conditions and maLPA_1_ mice subjected to the EPM). However, restraint stress exerted a far greater effect on WT mice than on maLPA_1_-null mice (*p* ≤ 0.01 WT mice compared with maLPA_1_-null mice) (Figure 5A). Restraint stress also modified the concentrations of LPA species. Thus, this procedure induced a significant increase in the 18:0 LPA concentration in both WT and maLPA_1_-null mice compared with the control group and the group of mice in which stress was induced by the EPM. However, in the WT animals, a significantly greater effect of restraint stress was observed than in maLPA_1_-null mice (LSD: *p* ≤ 0.05). An effect of stress on the concentration of 18:1 LPA was observed [stress effect: *F*(2, 26) = 21.45; *p* ≤ 0.0005]. The LPA 16:0 concentration was also affected by restraint stress, but only in WT animals, which showed a significantly higher level of this species than maLPA_1_-null mice. (LSD: *p* ≤ 0.001). In both WT and maLPA_1_-null mice, the EPM led to significant alterations in the 18:1 LPA concentration (LSD: *p* ≤ 0.05) (Figure 5B).

Considering the normalized lipid concentration, no interaction effects (genotype x stress) were observed on any of species examined. Stress affected the normalized lipid concentrations of both 18:1 [*F*(2, 24) = 10.85; *p* ≤ 0.005] and 18:2 LPA species [*F*(2, 24) = 3.42; *p* ≤ 0.05] (Figure 5C,D).

### CORT Levels

**FIGURE 6:**
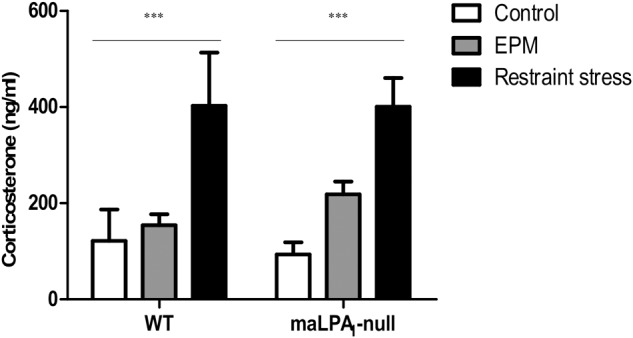
Corticosterone levels in WT and maLPA1-null mice. No differences were observed between WT and maLPA1-null mice under any of three environmental conditions. Restraint stress induced a significant increase in CORT levels in both genotypes. ^∗∗∗^*p* ≤ 0.0005 restraint stress compared with the control and EPM.

The analysis of plasma corticosterone levels did not reveal any significant differences between genotypes at baseline, after EPM and after restraint stress. However, in WT and maLPA_1_-null mice, restraint stress significantly increased corticosterone levels [*F*(2,27) = 13.62; *p* ≤ 0.0005; LSD *p* ≤ 0.005 for the comparisons of restraint stress with the control and EPM] (Figure 6A).

### Relationship Between LPA Species and EPM Outcomes

**FIGURE 7:**
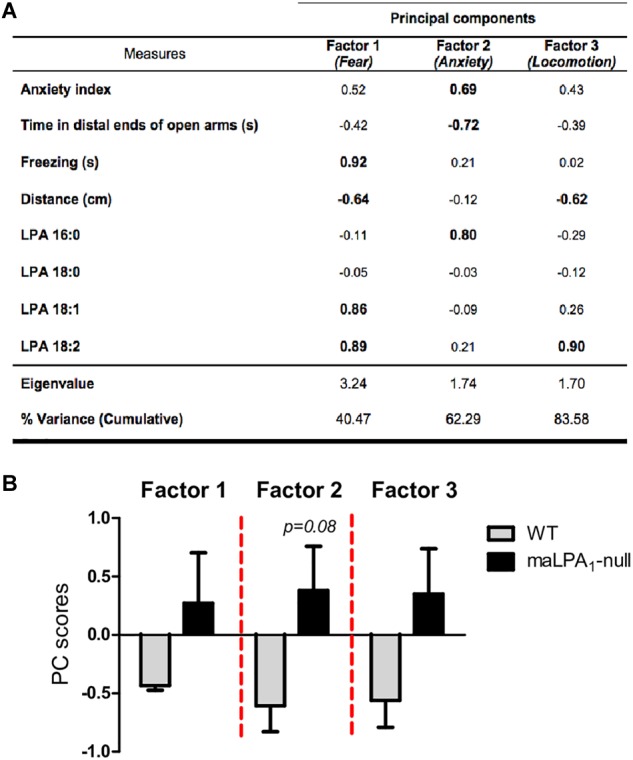
**(A)** Principal component analysis of anxious behaviors and measures of LPA species. Interpretable factor loading values are shown in bold. Variables with a negative score are inversely correlated with the factor. Rotation method: varimax with Kaiser normalization. The rotation converged in 3 iterations. KMO = 0.53; χ = 63.07; *p* ≤ 0.001. **(B)** Factor scores were calculated and compared between genotypes. PC scores were calculated by regression method in the principal component analysis and represent the loading in each factor. The maLPA_1_-null mice showed a higher score for the anxiety factor than WT mice (*p* = 0.08).

A PCA was performed to study the relationships among the hippocampal LPA species and behaviors exhibited by animals in the EPM (Figure 7). PCA with variance-maximizing (varimax) rotation revealed a 3-component solution accounting for 83% of the total variance. Strong positive correlation between the LPA 18:0 and 18:1 levels with freezing behaviors and negative correlations with distance traveled (factor 1, which was named “fear”) were observed. A positive correlation was observed between the levels of 16:0 LPA with the anxiety index and a negative correlation with time spent in the distal end (factor 2, which was named anxiety). Finally, the levels of 18:0 LPA were negatively correlated with distance (factor 3, which was named exploration) (Figure 7A). Not surprisingly, the distance traveled exhibited correlations in two factors. In this sense, increased freezing behavior, which indicates increased fear, is related to reduced locomotion. Moreover, the distance traveled is an important parameter of locomotion. Finally, factor scores were calculated and compared between genotypes. The maLPA_1_-null mice showed more fear and anxiety and less exploration than WT mice, but only a tendency toward significance was observed in factor 2 (anxiety) (t_11_ = 1.92; *p* = 0.08) (Figure 7B).

## Discussion

Here, we have characterized the effects of genetic manipulation and exposure to different environmental stresses, such as EPM and restraint stress, on hippocampal levels of LPA species. The classical version of EPM or restraint were used to determine the effect of moderate or more intense acute stress on the hippocampal LPA profile. Additionally, the possible roles of different hippocampal LPA species in behavior have been determined.

Lysophosphatidic acid is an important signaling molecule in the brain that is involved in multiple physiological actions and pathophysiological conditions (Fukushima et al., 2001; Tigyi and Parrill, 2003; Aoki, 2004; Ishii et al., 2004). The biological functions of LPA species are associated with specific G protein-coupled receptors named LPA_1-6_ (Choi and Chun, 2013). These receptors are expressed in the brain and may be involved in emotional behavior, particularly the receptors LPA_1_ (Santin et al., 2009; Castilla-Ortega et al., 2011; Pedraza et al., 2014; Moreno-Fernández et al., 2017, 2018a,b); LPA_2_ (Schneider et al., 2018); LPA_3_ (Uchida et al., 2014; Ueda et al., 2018) and LPA_5_ (Callaerts-Vegh et al., 2012; Tsukahara et al., 2018). The involvement on emotional regulation of the LPA_1_ receptor, through which lysophosphatidic acid signals, has been the most widely investigated (Santin et al., 2009; Castilla-Ortega et al., 2011; Pedraza et al., 2014; Moreno-Fernández et al., 2017, 2018a,b). LPA species containing both saturated and unsaturated long chain fatty acids (e.g., C16 and C18) are able to activate LPA_1_ receptors (Bandoh et al., 2000). However, surprisingly, the LPA_1_ receptor deficiency did not induce gross perturbations in the hippocampal LPA concentrations. In contrast to LPA_3_-null mice (Ma et al., 2009), “*de novo”* production of LPA (or converted LPA) was not absent in LPA_1_-null mice. In fact, the hippocampal LPA levels in maLPA_1_-null mice were similar to those observed in WT animals. The LPA_3_ receptor is an important determinant of LPA synthesis (Ma et al., 2009). The LPA_2_ receptor, although to a lesser extent, might also contribute to LPA production (Zhao et al., 2009). However, at least in the hippocampus, the LPA_1_ receptor does not seem to play a crucial role in LPA synthesis. However, since the hippocampal LPA pattern is slightly modified in maLPA_1_-null mice, with a significant increase in the concentration of saturated 18:0 LPA, a reasonable hypothesis is that the LPA_1_ receptor deficiency may modify the catalytic activity of phospholipase A_1_ (PLA_1_) and phospholipase A_2_ (PLA_2_), which are responsible for producing saturated LPA species. However, other pathways have not been excluded. Undoubtedly, the LPA_1_ and LPA_3_ receptors may have different key roles in the amplification of LPA production (Ma et al., 2009), based on the data obtained from measurements of LPA concentrations following the deletion of both the LPA_1_ and LPA_3_ receptors and supported by the findings from the present study in maLPA_1_-null mice.

However, stress, particularly the most intense protocol of stress, i.e., restraint, increased the total hippocampal LPA concentration and produced profound changes in LPA species, revealing that hippocampal LPA is a key target of stress. Thus, although EPM increased the concentration of the 18:2 LPA species, restraint stress substantially affected all the LPA species examined. For this reason, the profile was not altered after restraint stress; however, the EPM modified the LPA pattern in the hippocampus. In this group, the percentage of the relative abundance of 18:2 LPA was significantly higher than the control group. The effects of stress were also observed on maLPA_1_-null mice in the same direction as WT animals. However, the changes were less dramatic in maLPA_1_-null mice than in WT animals. Because control mice remained undisturbed in their home cages and unlike stressed animals, were not exposed to handling, which most likely is stressful, enable us to isolate the effect of stress in hippocampal LPA profile. On the other hand, although the forced exposure to open arms, a good procedure to study the effect of acute stress in mice (Neumann et al.), the classic version of EPM, is a more appropriate procedure to determine the effect of moderate acute stress on the hippocampal LPA profile in maLPA1-null mice because the behavioral response exhibited by the animals of this genotype in aversive test is exaggerated, similar to the panic-like reaction (Pedraza et al., 2014; Moreno-Fernández et al., 2017).

Because chronic stress alters the expression of lipids modifying enzyme in brain regions susceptible to the negative effects of stress (Patel et al., 2009), we postulated that the increased hippocampal levels of LPA induced by restraint stress may be due to changes in the expression and/or activity of autotaxin (ATX), which strongly correlate with LPA concentrations (Watanabe et al., 2007; Nakamura et al., 2008). Some of the most consistent consequences of stress are altered activity of the hypothalamus-pituitary-adrenal (HPA) axis and increased CORT levels (Sapolsky et al., 1985; McEwen et al., 1999; Kofman, 2002; Boettcher et al., 2017). A significant increase in CORT levels was observed in both genotypes after restraint stress, although, surprisingly, no differences were observed between genotypes after exposure to acute stress, as previously described (Pedraza et al., 2014). Differences in the experimental procedure probably explain the apparent inconsistencies between studies, (i.e., 30 vs. 60 min of restraint and 0 vs. 10 min elapsed between restraint stress and blood collection). Plasma corticosterone levels progressively decrease over time after restraint stress (Uchida et al., 2010; Shafiei et al., 2012). Because the addition of dexamethasone, a synthetic analog of CORT, to cells in culture increases the levels of the ATX mRNA and protein, and secretion of this enzyme (Honjo et al., 2018), an increase in CORT levels may increase ATX levels and its enzymatic activity. Moreover, after the EPM, the hippocampal LPA profile, but not the LPA levels, was modified. Although ATX may be involved in the mechanism underlying the observed differences, no changes in CORT levels were observed in animals subjected to the EPM and thus an additional pathway might also be involved (Tanaka et al., 2006; Van Meeteren et al., 2006) and may explain the limited increase in LPA levels in WT mice compared with restraint stress.

Additional studies are needed to clarify the underlying mechanisms responsible for the increase in LPA concentrations after restraint stress and the modified profile induced by the EPM. Undoubtedly, metabolic alterations in hippocampal LPA species occur after acute stress, particularly restrain stress, leading to a cascade of molecular events within this stress-responsive brain region. One of this events critical for proper control of the stress system is the balance between excitatory and inhibitory inputs (Kim, et al., 2016). Because LPA is involved in a proper excitation-inhibition balance (García-Morales et al., 2015), an increase in the concentrations of LPA may be involved in the translation of stress effects.”

As expected, the LPA_1_ receptor deficiency conferred an anxiogenic phenotype in the EPM (Santin et al., 2009; Castilla-Ortega et al., 2010; Moreno-Fernández et al., 2017, 2018a). As observed in previous studies, an increased anxiety index and low activity (Santin et al., 2009; Castilla-Ortega et al., 2010), together with a lower percentage of maLPA_1_-null mice (62% vs. 100% of WT) that entered the distal ends of the open arms and less time spent in the open arms and in the distal end indicates enhanced anxious-like behaviors. Coincidentally, maLPA_1_-null mice exhibited increased freezing, that may explain the reduced locomotor activity, and risk assessment behaviors and reduced rearing and head dipping behaviors that may indicate emotional hyperreactivity (Pedraza et al., 2014; Moreno-Fernández et al., 2017, 2018a). However, while the participation of the LPA_1_ receptor in anxiety is an established fact, researchers have not determined whether relationships exist among LPA species and anxiety parameters. Moreover, few insights into the effects of the administration of LPA or an agonist of LPA receptors on mood-related impairments have been obtained (Castilla-Ortega et al., 2014; Kim et al., 2017), and the evidence is inconclusive. Therefore, a pertinent question may be whether the behavioral performance in emotion-related tasks, such as the EPM, is associated with changes in the hippocampal concentrations of LPA species. As described above, the EPM modified the hippocampal LPA profile. Therefore, a PCA was performed to study the possible relationships among hippocampal LPA species and behavioral performance on the EPM. Fear behaviors were related to the hippocampal levels of LPA 18:1 and 18:2. Interestingly, these species were also the two hippocampal species whose percentage of relative abundance exhibited the greatest changes after the EPM. Anxiety was related to LPA 16:0 levels. In contrast, locomotion was associated with the concentration of 18:0 LPA species. However, unsurprisingly, the distance load in the two-factor analysis (1 and 3) was altered, because freezing behavior also has a locomotor component. Increased freezing involved reduced movement and was associated with the concentrations of 18:1 and 18:2 LPA. Validation in pharmacological studies is necessary to assess the contributions of the hippocampal LPA species to the regulation of emotion. Only one study may provide insights into the role of the 18:1 LPA species in behavior. Thus, although our observations are not completely comparable with previous studies, since the administration of LPA 18:1 to rats induced hypolocomotion in the open field (Castilla-Ortega et al., 2014), the levels of this LPA species may be important for controlling locomotion and high emotional arousal. Further studies are needed to determine whether changes in LPA species explain some components of emotional behavior. Moreover, the low N used in the present study is a limitation. Future research increasing number of animals and using different experimental approaches may provide stronger evidence of causality between changes in LPA species and behavior.

Nevertheless, the present study provides insights into the links between stress, anxiety and LPA species.

To the best of our knowledge, this study is the first to detect, identify and profile LPA species in the hippocampus of both LPA_1_-receptor null and WT mice. Moreover, the effect of acute stress on the hippocampal concentrations of LPA species and the possible relationships of these lipids with behavior have been explored for the first time. The LPA_1_ receptor deficiency did not disturb the levels of LPA species, but slightly modified the profile. In contrast, stress, particularly the most intense procedure used, significantly altered the hippocampal LPA levels. Finally, the emotional stress inherent in the EPM modified the LPA profile, and relationships among LPA species and behavioral outcomes were established, as the levels of 16:0 LPA in the hippocampus were associated with anxiety, 18:1 and 18:2 LPA levels were associated with fear and 18:0 levels were associated with locomotion. The hippocampal LPA species may be a key pharmacological target to mitigate the effects of stress on the hippocampus and emotional behaviors that are involved in psychopathological conditions, such as anxiety.

## Ethics Statement

Procedures were approved by the Ethics Committee of Malaga University (CEUMA 2013-0008-A/CEUMA: 1-2015- A, 08-7- 15-273) and Junta of Andalucia (08-7-15-273) and were performed in compliance with European animal research laws [European Communities Council Directives 2010/63/UE, 90/219/CEE, Regulation (EC) No. 1946/2003] and Spanish National and Regional Guidelines for Animal Experimentation and Use of Genetically Modified Organisms (Real Decreto 53/2013, Ley 32/2007, and Ley 9/2003, Real Decreto 178/2004, and Decreto 320/2010).

## Author Contributions

ST performed the lipid determination and was supervised by TO, FRdF, and GE-T. RM-F, EZ-I, and AN-Q performed the animal studies and were supervised by LS and GE-T. RM-F, ST, and AN-Q performed the molecular determination and were supervised by MG-F. ST and RM-F performed the statistical analyses and were supervised by LS and CP. JC performed the validation study. TO, FRdF, LS, MP-M, and CP contributed to interpreting the results. MP-M and CP designed the experiments, supervised the progression of the study and drafted the manuscript. All authors read and approved the final manuscript.

## Conflict of Interest Statement

The authors declare that the research was conducted in the absence of any commercial or financial relationships that could be construed as a potential conflict of interest.

## References

[B1] Aikawa S. Hashimoto K. Kano K. Aoki J. (2015). JB special review—recent progress in lipid mediators lysophosphatidic acid as a lipid mediator with multiple biological actions. *J. Biochem.* 2015 81–89. 10.1093/jb/mvu077 25500504

[B2] Aoki J. (2004). Mechanisms of lysophosphatidic acid production. *Semin. Cell Dev. Biol.* 15 477–489. 10.1016/j.semcdb.2004.05.001 15271293

[B3] Balaban C. D. Ogburn S. V. Warshafsky S. G. Ahmed A. Yates B. J. (2014). Identification of neural networks that contribute to motion sickness through principal components analysis of fos labeling induced by galvanic vestibular stimulation. *PLoS One* 9:e86730. 10.1371/journal.pone.0086730 24466215 PMC3900607

[B4] Bandoh K. Aoki J. Taira A. Tsujimoto M. Arai H. Inoue K. (2000). Lysophosphatidic acid (LPA) receptors of the EDG family are differentially activated by LPA species. Structure-activity relationship of cloned LPA receptors. *FEBS Lett.* 478 159–165. 10.1016/S0014-5793(00)01827-5 10922489

[B5] Bibancos T. Jardim D. L. Aneas I. Chiavegatto S. (2007). Social isolation and expression of serotonergic neurotransmission-related genes in several brain areas of male mice. *Genes Brain Behav.* 6 529–539. 10.1111/j.1601-183X.2006.00280.x 17083332

[B6] Boettcher C. Hartmann M. F. Zimmer K. P. Stefan A. W. (2017). High glucocorticoid response to 24-h-shift stressors in male but not in female physicians. *Front. Endocrinol.* 8:171. 10.3389/fendo.2017.00171 28769874 PMC5513946

[B7] Bou Khalil M. Hou W. Zhou H. Elisma F. Swayne L. A. Blanchard A. P. (2010). Lipidomics era: accomplishments and challenges. *Mass Spectrom. Rev.* 29 877–929. 10.1002/mas.20294 20931646

[B8] Callaerts-Vegh Z. Leo S. Vermaercke B. Meert T. D’Hooge R. (2012). LPA 5 receptor plays a role in pain sensitivity, emotional exploration and reversal learning. *Genes Brain Behav.* 11 1009–1019. 10.1111/j.1601-183X.2012.00840.x 23039190

[B9] Carobrez A. P. Bertoglio L. J. (2005). Ethological and temporal analyses of anxiety-like behavior: the elevated plus-maze model 20 years on. *Neurosci. Biobehal. Rev.* 29 1193–1205. 10.1016/J.NEUBIOREV.2005.04.017 16084592

[B10] Castilla-Ortega E. Escuredo L. Bilbao A. Pedraza C. Orio L. Estivill-Torrús G. (2014). 1-Oleoyl lysophosphatidic acid: a new mediator of emotional behavior in rats. *PLoS One* 9:e85348. 10.1371/journal.pone.0085348 24409327 PMC3883702

[B11] Castilla-Ortega E. Hoyo-Becerra C. Pedraza C. Chun J. Rodríguez De Fonseca F. Estivill-Torrús G. (2011). Aggravation of chronic stress effects on hippocampal neurogenesis and spatial memory in LPA1 receptor knockout mice. *PLoS One* 6:e25522. 10.1371/journal.pone.0025522 21980482 PMC3183048

[B12] Castilla-Ortega E. Pavón F. J. Sánchez-Marín L. Estivill-Torrús G. Pedraza C. Blanco E. (2016). Both genetic deletion and pharmacological blockade of lysophosphatidic acid LPA1 receptor results in increased alcohol consumption. *Neuropharmacology* 103 92–103. 10.1016/j.neuropharm.2015.12.010 26700247

[B13] Castilla-Ortega E. Sánchez-López J. Hoyo-Becerra C. Matas-Rico E. Zambrana-Infantes E. Chun J. (2010). Exploratory, anxiety and spatial memory impairments are dissociated in mice lacking the LPA1 receptor. *Neurobiol. Learn. Mem.* 94 73–82. 10.1016/j.nlm.2010.04.003 20388543 PMC3684252

[B14] Choi J. W. Chun J. (2013). Lysophospholipids and their receptors in the central nervous system. *Biochim. Biophys. Acta* 1831 20–32. 10.1016/j.bbalip.2012.07.015 22884303 PMC3693945

[B15] Choi J. W. Herr D. R. Noguchi K. Yung Y. C. Lee C. W. Mutoh T. (2010). LPA receptors: subtypes and biological actions. *Annu. Rev. Pharmacol. Toxicol.* 50 157–186. 10.1146/annurev.pharmtox.010909.105753 20055701

[B16] Chun J. Hla T. Wouter M. Sarah S. (2013). *Lysophospholipid Receptors: Signaling and Biochemistry*. Hoboken, NJ: John Wiley & Sons, Inc 10.1002/9781118531426

[B17] Cohen H. Kaplan Z. Matar M. A. Buriakovsky I. Bourin M. Kotler M. (2004). Different pathways mediated by CCK1 and CCK2 receptors: effect of intraperitoneal mrna antisense oligodeoxynucleotides to cholecystokinin on anxiety-like and learning behaviors in rats. *Depress. Anxiety* 20 139–152. 10.1002/da.20032 15487014

[B18] Contos J. J. A. Fukushima N. Weiner J. A. Kaushal D. Y. Chun J. (2000). Requirement for the lp A1 lysophosphatidic acid receptor gene in normal suckling behavior. *Proc. Natl. Acad. Sci. U.S.A* 97 13384–13389. 10.1073/pnas.97.24.13384 11087877 PMC27233

[B19] Contreras C. M. Rodríguez-Landa J. F. García-Ríos R. I. Cueto-Escobedo J. Guillen-Ruiz G. Bernal-Morales B. (2014). Myristic acid produces anxiolytic-like effects in wistar rats in the elevated plus maze. *Biomed. Res. Int.* 2014 1–8. 10.1155/2014/492141 25328885 PMC4189847

[B20] da Costa Estrela D. da Silva M. A. Guimarães A. T. de Oliveira Mendes M. da Silva Castro A. L. da Silva Torres I. L. (2015). Predictive behaviors for anxiety and depression in female wistar rats subjected to cafeteria diet and stress. *Physiol. Behav.* 151 252–263. 10.1016/j.physbeh.2015.07.016 26241160

[B21] DeMar J. C. Ma K. Bell J. M. Igarahsi M. Greenstein D. Rapoport S. I. (2006). One generation of n-3 polyunsaturated fatty acid deprivation increase depression and aggression test scores in rats. *J. Lipid Res.* 47 1052–1065. 10.1194/jlr.M500362-JLR200 16210728

[B22] Estivill-Torrus G. Llebrez-Zayas P. Matas-Rico E. Santin L. J. Pedraza C. De Diego I. (2008). Absence of LPA1 signaling results in defective cortical development. *Cereb. Cortex* 18 938–950. 10.1093/cercor/bhm132 17656621

[B23] Faria N. T. Marques S. Fonseca C. Ferreira F. C. (2015). Direct xylan conversion into glycolipid biosurfactants, mannosylerythritol lipids, by pseudozyma antarctica PYCC 5048(T). *Enzyme Microb. Technol.* 71 58–65. 10.1016/j.enzmictec.2014.10.008 25765311

[B24] Filgueiras G. B. Carvalho-Netto E. F. Estanislau C. (2014). Aversion in the elevated plus-maze: role of visual and tactile cues. *Behav. Process.* 107 106–111. 10.1016/j.beproc.2014.08.005 25151938

[B25] Fodor A. Kovács K. B. Balázsfi D. Klausz B. Pintér O. Demeter K. (2016). Depressive and anxiety-like behaviors and stress-related neuronal activation in vasopressin-deficient female brattleboro rats. *Physiol. Behav.* 158 100–111. 10.1016/j.physbeh.2016.02.041 26939727

[B26] Fukushima N. Ishii I. Contos J. J. Weiner J. A. Chun J. (2001). Lysophospholipid receptors. *Annu. Rev. Pharmacol. Toxicol.* 41 507–534. 10.1146/annurev.pharmtox.41.1.507 11264467

[B27] García-Fernández M. Castilla-Ortega E. Pedraza C. Blanco E. Hurtado-Guerrero I. Barbancho M. A. (2012). Chronic immobilization in the malpar1 knockout mice increases oxidative stress in the hippocampus. *Int. J. Neurosci.* 122 583–589. 10.3109/00207454.2012.693998 22591409

[B28] García-Morales V. Montero F. González-Forero D. Rodríguez-Bey G. Gómez-Pérez L. Medialdea-Wandossell M. J. (2015). Membrane-derived phospholipids control synaptic neurotransmission and plasticity. *PLoS Biol.* 13:e1002153. 10.1371/journal.pbio.1002153 25996636 PMC4440815

[B29] Hayashi K. Takahashi M. Nishida W. Yoshida K. Ohkawa Y. Kitabatake A. (2001). Phenotypic modulation of vascular smooth muscle cells induced by unsaturated lysophosphatidic. *Acids Circ. Res.* 89 251–258. 10.1161/hh1501.094265 11485975

[B30] Honjo M. Igarashi N. Nishida J. Kurano M. Yatomi Y. Igarashi K. (2018). Role of the autotaxin-LPA pathway in dexamethasone-induced fibrotic responses and extracellular matrix production in human trabecular meshwork cells. *Invest. Ophthalmol. Vic. Sci.* 59 21–30. 10.1167/iovs.17-22807 29305605

[B31] Horrobin D. F. Bennett C. N. (1999). Depression and bipolar disorder: relationships to impaired fatty acid and phospholipid metabolism and to diabetes, cardiovascular disease, immunological abnormalities, cancer, ageing and osteoporosis possible candidate genes. *Prostaglandins Leukots. Essent. Fatty Acids* 60 217–234. 10.1054/plef.1999.0037 10397403

[B32] Ishii I. Fukushima N. Ye X. Chun J. (2004). Lysophospholipid receptors: signaling and biology. *Annu. Rev. Biochem.* 73 321–354. 15189145 10.1146/annurev.biochem.73.011303.073731

[B33] Kihara Y. Mizuno H. Chun J. (2015). Lysophospholipid receptors in drug discovery. *Exp. Cell. Res.* 333 171–177. 10.1016/j.yexcr.2014.11.020 25499971 PMC4408218

[B34] Kim H. J. Park S. D. Lee R. M. Choi S. H. Hwang S. H. Rhim H. (2017). Gintonin attenuates depressive-like behaviors associated with alcohol withdrawal in mice. *J. Affect. Disord.* 215 23–29. 10.1016/j.jad.2017.03.026 28314177

[B35] Kofman O. (2002). The role of prenatal stress in the etiology of developmental behavioural disorders. *Neurosci. Biovehav. Rev.* 26 457–470. 10.1016/S0149-7634(02)00015-5 12204192

[B36] Ladrón de Guevara-Miranda D. Moreno-Fernández R. D. Gil-Rodríguez S. Rosell-Valle C. Estivill-Torrús G. Serrano A. (2019). Lysophosphatidic acid-induced increase in adult hippocampal neurogenesis facilitates the forgetting of cocaine-contextual memory. *Addict. Biol.* 24 458–470. 10.1111/abd.12612 29480526

[B37] Lee L. H. Shui G. Farooqui A. A. Wenk M. R. Tan C. H. Ong W. Y. (2009). Lipidomic analyses of the mouse brain after antidepressant treatment: evidence for endogenous release of long-chain fatty acids? *Int. J. Neuropsychopharmacol.* 12 953–964. 10.1017/S146114570900995X 19203412

[B38] Lin M. E. Herr D. R. Chun J. (2010). Lysophosphatidic acid (LPA) receptors: signaling properties and disease relevance. *Prostaglandins Other Lipid Mediat.* 91 130–138. 10.1016/j.prostaglandins.2009.02.002 20331961 PMC2845529

[B39] Ma L. Nagai J. Chun J. Ueda H. (2013). An LPA species (18:1 LPA) plays key roles in the self-amplification of spinal LPA production in the peripheral neuropathic pain model. *Mol. Pain* 9:29. 10.1186/1744-8069-9-29 23773289 PMC3691926

[B40] Ma L. Uchida H. Nagai J. Inoue M. Chun J. Aoki J. (2009). Lysophosphatidic Acid-3 receptor-mediated feed-forward production of lysophosphatidic acid: an initiator of nerve injury-induced neuropathic pain. *Mol. Pain* 5:64. 10.1186/1744-8069-5-64 19912636 PMC2780384

[B41] Matas-Rico E. García-Diaz B. Llebrez-Zayas P. López-Barroso D. Santín L. J. Pedraza C. (2008). Deletion of lysophosphatidic acid receptor LPA1 reduces neurogenesis in the mouse dentate gyrus. *Mol. Cell. Neurosci.* 39 342–355. 10.1016/j.mcn.2008.07.014 18708146 PMC3667670

[B42] McEwen B. S. de Leon M. J. Lupien S. J. Meaney M. J. (1999). Corticosteroids, the aging brain and cognition. *Trends Endocrinol. Metab.* 10 92–96. 10.1016/S1043-2760(98)00122-2 10322401

[B43] Mendes-Gomes J. Miguel T. T. Cristiane V. Amaral S. Nunes-De-Souza R. L. (2011). Corticosterone does not change open elevated plus maze-induced antinociception in mice. *Horm. Behav.* 60 408–413. 10.1016/j.yhbeh.2011.07.004 21798262

[B44] Miranda A. M. Bravo F. V. Chan R. B. Sousa N. Di Paolo G. Oliveira T. G. (2019). Differential lipid composition and regulation along the hippocampal longitudinal axis. *Transl. Psychiatry* 9:144. 10.1038/s41398-019-0478-6 31028243 PMC6486574

[B45] Mirendil H. Thomas E. A. De Loera C. Okada K. Inomata Y. Chun J. (2015). LPA signaling initiates schizophrenia-like brain and behavioral changes in a mouse model of prenatal brain hemorrhage. *Transl. Psychiatry* 5:e541. 10.1038/tp.2015.33 25849980 PMC4462599

[B46] Moreno-Fernández R. D. Pérez-Martín M. Castilla-Ortega E. Rosell del Valle C. García-Fernández M. I. Chun J. (2017). MaLPA1-null mice as an endophenotype of anxious depression. *Transl. Psychiatry* 7:e1077. 10.1038/tp.2017.24 28375206 PMC5416683

[B47] Moreno-Fernández R. D. Nieto-Quero A. Gómez-Salas F. J. Chun J. Estivill-Torrús G. Rodríguez de Fonseca F. (2018). Effects of genetic deletion versus pharmacological blockade of the LPA1 receptor on depression-like behaviour and related brain functional activity. *Dis. Model. Mech.* 11:dmm035519. 10.1242/dmm.035519 30061118 PMC6177006

[B48] Moreno-Fernández R. D. Tabbai S. Castilla-Ortega E. Perez-Martin M. Estivill-Torrus G. Rodriguez de Fonseca F. (2018). Stress, depression, resilience and ageing: a role for the LPA-LPA1 pathway. *Curr. Neuropharmacol.* 16 271–283. 10.2174/1570159X15666170710200352 28699486 PMC5843979

[B49] Morishige J. Urikura M. Takagi H. Hirano K. Koike T. Tanaka T. (2010). A clean-up technology for the simultaneous determination of lysophosphatidic acid and sphingosine-1-phosphate by matrix-assisted laser desorption/ionization time-of-flight mass spectrometry using a phosphate-capture molecule, phos-tag. *Rapid Commun. Mass Spectrom.* 24 1075–1084. 10.1002/rcm.4484 20213695

[B50] Müller C. P. Reichel M. Muhle C. Rhein C. Gulbins E. Kornhuber J. (2015). Brain membrane lipids in major depression and anxiety disorders. *Biochim. Biophys. Acta* 1851 1052–1065. 10.1016/j.bbalip.2014.12 25542508

[B51] Nakamura K. Igarashi K. Ide K. Ohkawa R. Okubo S. Yokota H. (2008). Validation of an autotaxin enzyme immunoassay in human serum samples and its application to hypoalbuminemia differentiation. *Clin. Chim. Acta* 388 51–58. 10.1016/j.cca.2007.10.005 17963703

[B52] Nunes-de-Souza R. L. Canto-de-Souza A. Rodgers R. J. (2002). Effects of intra-hippocampal infusion of WAY-100635 on plus-maze behavior in mice: influence of site of injection and prior test experience. *Brain Res.* 927 87–96. 10.1016/S0006-8993(01)03335-2 11814435

[B53] Oka S. Ota R. Shima M. Yamashita A. Sugiura T. (2010). GPR35 is a novel lysophosphatidic acid receptor. *Biochem. Biophys. Res. Commun.* 395 232–237. 10.1016/j.bbrc.2010.03.169 20361937

[B54] Oliveira T. G. Chan R. B. Bravo F. V. Miranda A. Silva R. R. Zhou B. (2016). The impact of chronic stress on the rat brain lipidome. *Mol. Psychiatry* 21 80–88. 10.1038/mp.2015.14 25754084 PMC4565780

[B55] Ong K. L. Morris M. J. McClelland R. L. Maniam J. Allison M. A. Rye K. A. (2016). Lipids, lipoprotein distribution and depressive symptoms: the multi-ethnic study of atherosclerosis. *Transl. Psychiatry* 6:e962. 10.1038/tp.2016.232 27898070 PMC5290355

[B56] Orio L. Pavón F. J. Blanco E. Serrano A. Araos P. Pedraz M. (2013). Lipid transmitter signaling as a new target for treatment of cocaine addiction: new roles for acylethanolamides and lysophosphatidic acid. *Curr. Pharm. Des.* 19 7036–7049. 10.2174/1381611281940131209143421 23574441

[B57] Patel S. Kingsley P. J. Mackie K. Marnett L. J. Winder D. G. (2009). Repeated homotypic stress elevates 2-arachidonoylglycerol levels and enhances short-term endocannabinoid signaling at inhibitory synapses in basolateral amygdala. *Neuropsychopharmacology* 34 2699–2709. 10.1038/npp.2009.101 19675536 PMC2881681

[B58] Pedraza C. Sánchez-López J. Castilla-Ortega E. Rosell-Valle C. Zambrana-Infantes E. García-Fernández M. (2014). Fear extinction and acute stress reactivity reveal a role of LPA(1) receptor in regulating emotional-like behaviors. *Brain Struct. Funct.* 219 1659–1672. 10.1007/s00429-013-0592-9 23775489

[B59] Pellow S. Chopin P. File S. E. Briley M. (1985). Validation of open: closed arm entries in an elevated plus-maze as a measure of anxiety in the rat. *J. Neurosci. Methods* 14 149–167. 10.1016/0165-0270(85)90031-7 2864480

[B60] Rappley I. Myers D. S. Milne S. B. Ivanova P. T. LaVoie M. J. Brown H. A. (2009). Lipidomic profiling in mouse brain reveals differences between ages and genders, with smaller changes associated with α-synuclein genotype. *J. Neurochem.* 111 15–25. 10.1111/j.1471-4159.2009.06290.x 19627450 PMC2752313

[B61] Reis D. G. Scopinho A. A. Guimarães F. S. Corrêa F. M. Resstel L. B. (2011). Behavioral and autonomic responses to acute restraint stress are segregated within the lateral septal area of rats. *PLoS One* 6:e23171. 10.1371/journal.pone.0023171 21858017 PMC3156740

[B62] Sánchez-Marín L. Ladrón de Guevara-Miranda D. Mañas-Padilla M. C. Alén F. Moreno-Fernández R. D. Díaz-Navarro C. (2018). Systemic blockade of LPA1/3 lysophosphatidic acid receptors by ki16425 modulates the effects of ethanol on the brain and behavior. *Neuropharmacology* 133 189–201. 10.1016/j.neuropharm.2018.01.033 29378212

[B63] Sano T. Baker D. Viraq T. Wada A. YaAtomi Y. Kobayashi T. (2002). Multiple mechanisms linked to platelet activation result in lysophosphatidic acid and sphingosine 1-phosphate generation in blood. *J. Biol. Chem.* 14 21197–21206. 10.1074/jbc.M20128920 11929870

[B64] Santin L. J. Bilbao A. Pedraza C. Matas-Rico E. López-Barroso D. Castilla-Ortega E. (2009). Behavioral phenotype of MaLPA 1 -null mice: increased anxiety-like behavior and spatial memory deficits. *Genes Brain Behav.* 8 772–784. 10.1111/j.1601-183X.2009.00524.x 19689455 PMC4780438

[B65] Sapolsky R. M. Meaney M. J. McEwen B. S. (1985). The development of the glucocorticoid receptor system in the rat limbic brain. III. Negative-feedback regulation. *Brain Res.* 350 169–173. 10.1016/0165-3806(85)90261-5 3986611

[B66] Schneider P. Ho Y.-J. Spanagel R. Pawlak C. R. (2011). A novel elevated plus-maze procedure to avoid the one-trial tolerance problem. *Front. Behav. Neurosci.* 5:43. 10.3389/fnbeh.2011.00043 21845176 PMC3146044

[B67] Schneider P. Petzold S. Sommer A. Nitsch R. Schwegler H. Vogt J. (2018). Altered synaptic phospholipid signaling in PRG-1 deficient mice induces exploratory behavior and motor hyperactivity resembling psychiatric disorders. *Behav. Brain Res.* 336 1–7. 10.1016/j.bbr.2017.08.032 28843862

[B68] Shafiei N. Gray M. Viau V. Floresco S. B. (2012). Acute stress induces selective alterations in cost/benefit decision-making. *Neuropsychopharmacology* 37 2194–2209. 10.1038/npp.2012.69 22569506 PMC3422485

[B69] Sorregotti T. Mendes-Gomes J. Rico J. L. Rodgers R. J. Nunes-de-Souza R. L. (2013). Ethopharmacological analysis of the open elevated plus-maze in mice. *Behav. Brain Res.* 246 76–85. 10.1016/j.bbr.2013.02.035 23470900

[B70] Sugiura T. Kobayashi Y. Oka S. Waku K. (2002). Biosynthesis and degradation of anandamide and 2-arachidonoylglycerol and their possible physiological significance. *Protaglandins Leukot. Essent. Fatty Acids* 66 173–192. 10.1054/plef.2001.0356 12052034

[B71] Tabata K. Baba K. Shiraishi A. Ito M. Fujita N. (2007). The orphan GPCR GPR87 was deorphanized and shown to be a lysophosphatidic acid receptor. *Biochem. Biophys. Res. Commun.* 363 861–866. 10.1016/jbbrc.2007.09.063 17905198

[B72] Tabuchi S. (2015). The autotaxin-lysophosphatidic acid-lysophosphatidic acid receptor cascade: proposal of a novel potential therapeutic target for treating glioblastoma multiforme. *Lipids Health Dis.* 14:56. 10.1186/s12944-015-0059-5 26084470 PMC4477515

[B73] Tanaka M. Okudaira S. Kishi Y. Ohkawa R. Iseki S. Ota M. (2006). Autotaxin stabilizes blood vessels and is required for embryonic vasculature by producing lysophosphatidic acid. *J. Biol. Chem.* 281 25822–25830. 10.1074/jbc.M605142200 16829511

[B74] Tanaka T. Tsutsui H. Hirano K. Koike T. Tokumura A. Satouchi K. (2004). Quantitative analysis of lysophosphatidic acid by time-of-flight mass spectrometry using a phosphate-capture molecule. *J. Lipid Res.* 45 2145–2150. 10.1194/jlr.D400010-JLR200 15314093

[B75] Tigyi G. Parrill A. L. (2003). Molecular mechanisms of lysophosphatidic acid action. *Prog. Lipid Res.* 42 498–526. 10.1016/S0163-7827(03)00035-3 14559069

[B76] Trimbuch T. Beed P. Vogt J. Schuchmann S. Maier N. Kintscher M. (2009). Synaptic PRG-1 modulates excitatory transmission via lipid phosphate-mediated signaling. *Cell* 138 1222–1235. 10.1016/j.cell.2009.06.050 19766573 PMC3716297

[B77] Tsukahara R. Yamamoto S. Yoshikawa K. Gotoh M. Tsukahara T. Neyama H. (2018). LPA5 signaling is involved in multiple sclerosis-mediated neuropathic pain in the cuprizone mouse model. *J. Pharmacol. Sci.* 136 93–96. 10.1016/j.jphs.2018.01.001 29409686

[B78] Uchida H. Nagai J. Ueda H. (2014). Lysophosphatidic acid and its receptors LPA1 and LPA3 mediate paclitaxel-induced neuropathic pain in mice. *Mol. Pain* 10:71. 10.1186/1744-8069-10-71 25411045 PMC4246549

[B79] Uchida S. Hara K. Kobayashi A. Funato H. Hobara T. Otsuki K. (2010). Early life stress enhances behavioral vulnerability to stress through the activation of REST4-mediated gene transcription in the medial prefrontal cortex of rodents. *J. Neurosci.* 30 15007–15018. 10.1523/JNEUROSCI.1436-10.2010 21068306 PMC6633839

[B80] Ueda H. Neyama H. Sasaki K. Miyama C. Iwamoto R. (2018). Lysophosphatidic acid LPA1 and LPA3 receptors play roles in the maintenance of late tissue plasminogen activator-induced central poststroke pain in mice. *Neurobiol. Pain.* 5:100020 10.1016/J.YNPAI.2018.07.001 PMC6550111 31194070

[B81] Van Meeteren L. A. Ruurs P. Stortelers C. Bouwman P. van Rooijen M. A. Pradere J. P. (2006). Autotaxin, a secreted lysophospholipase D, is essential for blood vessel formation during development. *Mol. Cell. Biol.* 26 5015–5022. 10.1128/MCB.02419-05 16782887 PMC1489177

[B82] Violle N. Balandras F. Le Roux Y. Desor D. Schroeder H. (2009). Variations in illumination, closed wall transparency and/or extramaze space influence both baseline anxiety and response to diazepam in the rat elevated plus-maze. *Behav. Brain Res.* 203 35–42. 10.1016/j.bbr.2009.04.015 19389429

[B83] Walf A. A. Frye C. A. (2007). The use of the elevated plus maze as an assay of anxiety-related behavior in rodents. *Nat. Protoc.* 2 322–328. 10.1038/nprot.2007.44 17406592 PMC3623971

[B84] Watanabe N. Ikeda H. Nakamura K. Ohkawa R. Kume Y. Tomiya T. (2007). Plasma lysophosphatidic acid level and serum autotaxin activity are increased in liver injury in rats in relation to its severity. *Life Sci.* 81 1009–1015. 10.1016/j.lfs.2007.08.013 17850827

[B85] Yamada M. Tsukagoshi M. Hashimoto T. Oka J.-I. Saitoh A. Yamada M. (2015). Lysophosphatidic acid induces anxiety-like behavior via its receptors in mice. *J. Neural Transm.* 122 487–494. 10.1007/s00702-014-1289-9 25119538

[B86] Yoshida K. Nishida W. Hayashi K. Ohkawa Y. Ogawa A. Aoki J. (2003). Vascular remodeling induced by naturally occurring unsaturated lysophosphatidic acid in vivo. *Circulation* 108 1746–1752. 10.116/01.CIR.000089374 14504178

[B87] Yung Y. C. Stoddard N. C. Chun J. (2014). LPA receptor signaling: pharmacology, physiology, and pathophysiology. *J. Lipid Res.* 55 1214. 10.1194/jlr.R046458 24643338 PMC4076099

[B88] Zhao Y. Tong J. He D. Pendyala S. Evgeny B. Chun J. (2009). Role of lysophosphatidic acid receptor LPA 2 in the development of allergic airway inflammation in a murine model of asthma. *Respir. Res.* 10:114. 10.1186/1465-9921-10-114 19930563 PMC2788521

[B89] Zimprich A. Garrett L. Deussing J. M. Wotjak C. T. Fuchs H. Gailus-Durner V. (2014). A robust and reliable non-invasive test for stress responsivity in mice. *Front. Behav. Neurosci.* 8:125. 10.3389/fnbeh.2014.00125 24782732 PMC3995076

